# 4-Hexylresorcinol Attenuates Ethanol-Induced Hepatic and Pancreatic Injury by Modulating Metabolic Dysfunction and Endoplasmic Reticulum Stress

**DOI:** 10.3390/biomedicines14051077

**Published:** 2026-05-09

**Authors:** Horațiu Rotar, Soon-Young Kang, Hyun-Seung Kim, Seung-Ki Hong, Yoon-Jo Lee, Ji-Hyeon Oh, Suyeon Park, Jongho Choi, Xiangguo Che, Seong-Gon Kim, Je-Yong Choi

**Affiliations:** 1 Department of Oral and Maxillofacial Surgery and Implantology, Faculty of Dental Medicine, “Iuliu Hatieganu” University of Medicine and Pharmacy, 400012 Cluj-Napoca, Romania; dr.horatiu.rotar@gmail.com; 2 Department of Oral and Maxillofacial Surgery, College of Dentistry, Kangwon National University, Gangneung 25457, Republic of Korea; syk980208@naver.com (S.-Y.K.); dannyk0314@naver.com (H.-S.K.); weuuhh@gwnu.ac.kr (S.-K.H.); _yoonjo@gwnu.ac.kr (Y.-J.L.); 3 Department of Oral Pathology, College of Dentistry, Kangwon National University, 7 Jukheon-gil, Gangneung 25457, Republic of Korea; sypark9101@gwnu.ac.kr (S.P.); jhchoi@gwnu.ac.kr (J.C.); 4 Department of Biochemistry and Cell Biology, Cell and Matrix Research Institute, School of Medicine, Kyungpook National University, Daegu 41944, Republic of Korea; xiangguo0622@naver.com (X.C.); jechoi@knu.ac.kr (J.-Y.C.)

**Keywords:** 4-hexylresorcinol, ethanol-induced injury, hepatic steatosis, pancreatic β-cell dysfunction, endoplasmic reticulum stress, GADD153 (CHOP), glucose homeostasis, oxidative stress, glycogen depletion

## Abstract

**Background/Objectives**: Chronic ethanol (EtOH) consumption is a major cause of metabolic dysfunction and multi-organ injury, particularly in the liver and pancreas. Because oxidative stress and endoplasmic reticulum (ER) stress are central mechanisms in both organs, this study evaluated the protective efficacy of 4-hexylresorcinol (4HR) against EtOH-induced hepato-pancreatic injury. **Methods**: Male C57BL/6J mice (6 weeks old) were assigned to four groups (*n* = 10/group): control, EtOH, EtOH + 4HR (5 mg/kg), and EtOH + 4HR (10 mg/kg). After a 1-week adaptation period, mice were fed a liquid EtOH diet for 5 weeks. Glucose tolerance, fasting glucose, serum insulin, and insulinogenic index were assessed. Liver and pancreas were analyzed by histology, immunohistochemistry, Western blotting, periodic acid-Schiff staining, Oil Red O staining, and malondialdehyde assay. **Results**: Chronic EtOH exposure impaired glucose homeostasis, reduced the insulinogenic index, increased hepatic inflammation and ALT levels, depleted hepatic glycogen, elevated pancreatic lipid peroxidation, and upregulated GADD153 (CHOP) expression in both the liver and pancreas. 4HR administration, particularly at 10 mg/kg, attenuated several of these alterations. 4HR treatment was associated with reduced hepatic inflammatory changes and ALT elevation, decreased pancreatic malondialdehyde levels, and suppressed GADD153 expression in both organs. Although PAS staining in the 4HR-treated group showed a qualitative tendency toward increased hepatic glycogen deposition, quantitative analysis did not demonstrate significant recovery relative to the EtOH group. **Conclusions**: 4HR showed protective effects against several aspects of chronic EtOH-induced hepatic and pancreatic injury, including hepatic inflammation, pancreatic lipid peroxidation, and ER stress-related GADD153 expression. However, quantitative PAS analysis did not support significant restoration of EtOH-induced hepatic glycogen depletion by 4HR. These findings suggest that 4HR may serve as a potential multi-organ protective agent against alcohol-induced inflammatory, oxidative stress-, and ER stress-related injury, although its effect on hepatic glycogen metabolism remains limited under the present experimental conditions.

## 1. Introduction

Chronic ethanol (EtOH) consumption remains a major cause of metabolic and organ dysfunction worldwide. The liver, as the primary site of EtOH metabolism, undergoes significant oxidative stress initiated by alcohol dehydrogenase and cytochrome P450 2E1 (CYP2E1)-mediated oxidation. This process generates high levels of acetaldehyde and reactive oxygen species (ROS) [1,2]. The resulting oxidative burden triggers lipid peroxidation, compromises mitochondrial integrity, and activates pro-inflammatory pathways within hepatocytes [3,4]. These processes disrupt normal hepatic metabolism, resulting in triglyceride accumulation, glycogen depletion, and hepatocellular injury that can progress from steatosis to steatohepatitis and fibrosis [5]. In addition, EtOH metabolism alters the intracellular redox state by increasing the NADH/NAD^+^ ratio, which suppresses gluconeogenesis and promotes de novo lipogenesis, thereby exacerbating the metabolic imbalance in the liver [6].

EtOH-induced injury is not limited to the liver. The pancreas is also highly susceptible to EtOH-mediated toxicity. Chronic EtOH exposure induces oxidative stress and mitochondrial dysfunction in both pancreatic acinar and endocrine cells, leading to cellular injury and inflammatory responses. Elevated EtOH metabolites and oxidative stress have been shown to impair insulin secretion from pancreatic β-cells, thereby disrupting glucose homeostasis [7,8]. Consequently, chronic alcohol intake is associated with pancreatic inflammation, impaired endocrine function, and increased risk of metabolic disorders [9]. These observations indicate that EtOH exposure causes multi-organ metabolic injury affecting both the liver and pancreas through shared mechanisms involving oxidative stress and bioenergetic failure.

Because oxidative stress and metabolic dysregulation are central features of EtOH-induced organ injury, various pharmacological and nutritional approaches have been investigated to mitigate these effects. Antioxidants, including N-acetylcysteine [10], vitamin E [11], and various polyphenols [12], have been explored for their ability to reduce ROS-mediated damage. Other therapeutic strategies include agents that improve mitochondrial function, modulate lipid metabolism, or enhance cellular antioxidant defenses [13].

Despite these efforts, effective pharmacological interventions for EtOH-induced liver and pancreatic injury remain limited. Many antioxidant agents show protective effects in experimental models but demonstrate inconsistent efficacy in clinical settings [14,15]. Moreover, existing therapeutics focus primarily on hepatoprotection, overlooking the parallel dysfunction of the pancreas. Therefore, the identification and development of novel bifunctional compounds capable of concurrently protecting both hepatic and pancreatic tissues against EtOH-induced oxidative stress and metabolic insults represent a critical unmet clinical need.

4-Hexylresorcinol (4HR) is a phenolic compound that has been widely used as an antimicrobial and antiseptic agent in food preservation and medical applications [16,17]. Recent evidence, however, suggests that 4HR possesses antioxidant and anti-inflammatory properties [18]. As a phenolic derivative, 4HR can scavenge ROS and mitigate oxidative stress–mediated cellular damage [19]. Previous studies have also indicated that 4HR can modulate cellular stress responses and metabolic signaling pathways, including pathways associated with mitochondrial function and cellular energy metabolism [20]. Pancreatic β-cells are particularly vulnerable to oxidative stress because they possess relatively low levels of endogenous antioxidant enzymes [21]. In experimental models using streptozotocin (STZ), a diabetogenic compound that selectively damages pancreatic β-cells through ROS generation and DNA alkylation, oxidative stress leads to marked pancreatic tissue damage and hyperglycemia [22,23,24].

Interestingly, our recent study showed that 4HR attenuated streptozotocin-induced pancreatic β-cell damage and preserved insulin secretion, suggesting that 4HR may protect metabolically vulnerable tissues from oxidative stress–mediated injury. Because oxidative stress and ER stress are common mechanisms underlying ethanol-induced damage in both the liver and pancreas, we hypothesized that 4HR may exert protective effects across the hepato-pancreatic axis in chronic EtOH exposure. Therefore, the objective of the present study was to evaluate whether 4HR attenuates EtOH-induced hepatic and pancreatic injury in a chronic ethanol-feeding mouse model. To address this, we assessed glucose homeostasis, hepatic injury and glycogen depletion, pancreatic endocrine alterations, oxidative stress, and ER stress-related responses. The significance of this study lies in identifying 4HR as a potential multi-organ protective agent that may simultaneously mitigate liver and pancreatic damage caused by chronic EtOH exposure, a therapeutic area that remains insufficiently explored.

## 2. Materials and Methods

### 2.1. Experimental Animals and Study Design

Male C57BL/6J mice, a commonly used inbred strain for metabolic and EtOH-feeding studies, were purchased from Samtako Bio Korea (Osan, Republic of Korea). The animals were 6 weeks old at the start of the experiment and had an initial body weight of approximately 25 g. Animals were housed under standard laboratory conditions with controlled temperature and humidity and maintained under a 12 h light/dark cycle. Mice were housed two per cage, and food intake was measured daily on a per-cage basis. Food and water were provided ad libitum unless otherwise specified. All animal procedures were performed in accordance with institutional guidelines for the care and use of laboratory animals and were approved by the Institutional Animal Care and Use Committee of the authors’ institution (GWNU-2025-19. Approved on 3 November 2025).

Mice were randomly assigned to four experimental groups (*n* = 10 per group): (1) control group, (2) EtOH group, (3) EtOH + low-dose 4HR group (4HR5), and (4) EtOH + high-dose 4HR group (4HR10). 4HR (Cat. No. 209465; Sigma-Aldrich, St. Louis, MO, USA; purity 98%) was used in this study. Mice in the low-dose and high-dose 4HR groups received 4HR at 5 mg/kg and 10 mg/kg, respectively, by daily subcutaneous injection throughout the 5-week EtOH-feeding period.

All animals underwent a 1-week training period for adaptation to a liquid diet system. After the adaptation period, mice in the EtOH-fed groups received an EtOH-containing liquid diet based on the Lieber–DeCarli chronic ethanol-feeding model (Cat. No. 710260; Dyets Inc., Bethlehem, PA, USA), whereas the control group received an isocaloric control liquid diet (Cat. No. 710027; Dyets Inc.) [25]. The EtOH diet was prepared according to the manufacturer’s instructions by adding 67 mL of 95% EtOH per liter of diet, and EtOH was introduced gradually during the adaptation phase before being maintained at the target concentration throughout the 5-week experimental period. Throughout the study, body weight was monitored weekly, while general health status and food intake were assessed daily (Figure S1).

### 2.2. Glucose Tolerance Test and Serum Insulin Measurement

To evaluate glucose metabolism and pancreatic endocrine function, a glucose tolerance test (GTT) was performed at the end of the experimental period. Glucose tolerance testing was performed as previously described with minor modifications [26]. Mice were fasted overnight prior to the test while maintaining free access to water. After the fasting period, baseline blood glucose levels were measured from tail vein blood samples.

Glucose was administered by intraperitoneal injection using a 50 wt% dextrose solution at a dose of 2 g/kg body weight. Blood glucose levels were measured at 15, 30, 60, and 120 min after glucose administration using a GreenDoctor glucometer (GC Pharma, Yongin, Republic of Korea). Glucose tolerance was assessed by comparing glucose levels at each time point and by calculating the area under the curve (AUC) of the glucose response.

To evaluate pancreatic β-cell function, serum insulin levels were measured during the glucose tolerance test. Small volumes of blood were collected from the tail vein at fasting and 30 min following glucose administration. Blood samples were centrifuged to obtain serum, which was stored at −80 °C until analysis. Serum insulin concentrations were determined using a Mouse Insulin ELISA kit (Cat. No. 10-1247-01; Mercodia AB, Uppsala, Sweden) according to the manufacturer’s instructions. Briefly, 10 μL of each sample was added to the wells, followed by 100 μL of enzyme conjugate, incubated for 2 h at room temperature on a plate shaker, washed, developed with TMB substrate, and read at 450 nm.

The insulinogenic index was calculated to assess glucose-stimulated insulin secretory capacity. It was defined as the ratio of the increment in serum insulin concentration to the increment in blood glucose concentration after glucose loading, according to the following formula:

$$Insulinogenic\;index=\frac{Insulin\;at\;30\;min-Insulin\;at\;0\;min}{Glucose\;at\;30\;\min-\;Glucose\;at\;0\;min}$$

### 2.3. Histological Analysis

At the time of sacrifice, liver and pancreas tissues were harvested and immediately fixed in 10% neutral-buffered formalin. Liver samples were consistently collected from the left lateral lobe in all animals to minimize inter-lobar sampling variability. Fixed tissues were dehydrated through graded EtOH solutions, cleared in xylene, and embedded in paraffin. Paraffin sections (5 μm thickness) were prepared using a microtome and mounted on glass slides for histological analysis. For general histological evaluation, tissue sections were stained with hematoxylin and eosin (H&E) according to standard protocols. Briefly, sections were deparaffinized in xylene, rehydrated through graded EtOH, stained with hematoxylin, and counterstained with eosin. After dehydration and mounting, stained sections were examined under a light microscope. Representative microscopic images were acquired using a BX51 light microscope equipped with a DP73 digital camera (Olympus, Tokyo, Japan) at ×200 magnification under identical illumination conditions. For quantitative or semiquantitative histologic assessment, three representative non-overlapping microscopic fields were analyzed per section. In H&E-stained liver sections, inflammation was graded semi-quantitatively according to the criteria shown in Table 1, adapted from the histologic scoring system described by Kleiner et al. [27]. All image analyses were performed in a blinded manner.

To evaluate hepatic lipid accumulation, Oil Red O staining was performed on selected liver samples. Because Oil Red O staining requires frozen sections and frozen tissues were not available for all specimens, this analysis was conducted on representative samples from each experimental group. Briefly, liver tissues were embedded in optimal cutting temperature compound and frozen. Cryosections (10 μm thickness) were prepared using a cryostat and mounted onto glass slides. Sections were fixed with 10% neutral-buffered formalin, rinsed with distilled water, and stained with Oil Red O working solution to visualize neutral lipid droplets. After staining, sections were counterstained with hematoxylin, washed, and mounted for microscopic examination. Images were captured using a light microscope under identical imaging conditions. Lipid accumulation was evaluated qualitatively by comparing staining patterns among groups, and the Oil Red O–positive area was quantified using digital image analysis. Quantitative analysis was performed on representative images obtained from the selected samples.

To evaluate hepatic glycogen content, Periodic Acid–Schiff (PAS) staining was performed. After deparaffinization and rehydration, sections were treated with periodic acid followed by incubation with Schiff reagent according to the manufacturer’s instructions. PAS-positive glycogen deposits appeared as magenta staining within hepatocyte cytoplasm. For semiquantitative analysis of hepatic glycogen deposition, PAS-stained sections were examined by digital image analysis using representative microscopic images captured under identical illumination and magnification conditions. The magenta index was calculated as the percentage of PAS-positive magenta-stained area relative to the total tissue area within the selected region of interest (ROI). Because PAS-positive magenta staining in hepatocyte cytoplasm primarily reflects glycogen deposition, this index was used as a semiquantitative indicator of hepatic glycogen content. For each specimen, three representative microscopic fields were selected and analyzed, and the mean value was used as the representative value for that animal. To ensure reliability, the image analysis results were reviewed and confirmed by a human observer blinded to the experimental groups. To confirm glycogen specificity, selected sections were subjected to diastase digestion prior to PAS staining. Tissue sections were incubated with α-amylase (diastase) solution at 37 °C to enzymatically degrade glycogen. Reduction or disappearance of PAS staining after diastase treatment was interpreted as evidence that the PAS-positive signal was primarily glycogen-derived.

### 2.4. Immunohistochemistry

To evaluate the expression of endocrine and stress-related proteins in pancreatic tissues, immunohistochemical staining was performed for insulin, glucagon, and GADD153 (CHOP). Paraffin-embedded pancreatic tissue sections (5 μm thickness) were deparaffinized in xylene and rehydrated through graded EtOH solutions. Antigen retrieval was performed by heating the sections in citrate buffer (pH 6.0). Endogenous peroxidase activity was blocked by incubation with 3% hydrogen peroxide.

After blocking with normal serum to prevent nonspecific binding, tissue sections were incubated overnight at 4 °C with primary antibodies against insulin (Cat. No. sc-8033, Santa Cruz Biotechnology, Santa Cruz, CA, USA), glucagon (Cat. No. sc-514592, Santa Cruz Biotechnology), and GADD153 (CHOP; Cat. No. sc-166682, Santa Cruz Biotechnology). All primary antibodies were used at a dilution of 1:100. After washing with phosphate-buffered saline (PBS), sections were incubated with the Dako REAL EnVision horseradish peroxidase detection system (Dako, Glostrup, Denmark) according to the manufacturer’s instructions.

Immunoreactivity was visualized using 3,3′-diaminobenzidine (DAB) as the chromogenic substrate, and sections were counterstained with hematoxylin. Stained sections were dehydrated, mounted, and examined under a light microscope. Representative images were captured for comparison among experimental groups. For semiquantitative analysis of GADD153 immunostaining, three representative areas were selected from each liver section and three representative islets of Langerhans were selected from each pancreatic section. The average staining intensity within the selected regions was measured using SigmaScan Pro version 5.0 (SPSS Inc., Chicago, IL, USA). Relative intensity values were expressed on a grayscale scale ranging from 0 to 255, where 0 indicates the lowest intensity and 255 indicates the highest intensity. The mean value from the three selected areas was used as the representative value for each specimen.

### 2.5. Measurement of Lipid Peroxidation

Lipid peroxidation in pancreatic tissue was evaluated by measuring malondialdehyde (MDA) levels using a TBARS Assay Kit (Cat. No. 10009055; Cayman Chemical, Ann Arbor, MI, USA) according to the manufacturer’s instructions and previously described analytical approach [28]. Briefly, pancreatic tissues were homogenized in an appropriate buffer and centrifuged to obtain clear supernatants. The samples were then reacted with thiobarbituric acid (TBA) under high-temperature acidic conditions to form an MDA–TBA adduct. The resulting chromogenic product was measured spectrophotometrically at 532 nm, and MDA concentrations were calculated using an MDA standard curve provided in the kit. MDA levels were normalized to tissue weight and expressed as nmol/g tissue.

### 2.6. Western Blot Analysis

Protein levels of glucagon, GRP78, and GADD153 (CHOP) in pancreatic tissue were analyzed by Western blotting. Pancreatic tissues were homogenized in ice-cold radioimmunoprecipitation assay (RIPA) buffer (iNtRON Biotechnology, Seongnam, Republic of Korea) containing a protease inhibitor cocktail (Thermo Scientific, Rockford, IL, USA), and the homogenates were centrifuged to obtain total protein lysates from the supernatants.

Protein concentrations were measured using a bicinchoninic acid (BCA) protein assay (Cat. No. 23225; Thermo Fisher Scientific, Rockford, IL, USA). Equal amounts of protein were separated by sodium dodecyl sulfate-polyacrylamide gel electrophoresis (SDS-PAGE) and transferred onto polyvinylidene difluoride (PVDF) membranes. The membranes were blocked with 5% nonfat dry milk and incubated overnight at 4 °C with primary antibodies against glucagon (Cat. No. sc-514592; Santa Cruz Biotechnology), GRP78 (Cat. No. sc-13539; Santa Cruz Biotechnology), and GADD153/CHOP (Cat. No. sc-166682; Santa Cruz Biotechnology). After washing, the membranes were incubated with horseradish peroxidase-conjugated secondary antibodies at a dilution of 1:10,000. Protein bands were detected using an enhanced chemiluminescence (ECL) detection system and visualized using a ChemiDoc imaging system (Bio-Rad, Hercules, CA, USA). GAPDH (Cat. No. sc-137179; Santa Cruz Biotechnology) and β-actin (Cat. No. sc-81178; Santa Cruz Biotechnology) were used as the internal loading control.

Band intensities were quantified using SigmaScan Pro version 5.0 (SPSS Inc., Chicago, IL, USA) and normalized to GAPDH and β-actin. Western blot experiments were performed in duplicate.

### 2.7. Statistical Analysis

All quantitative data are presented as mean ± standard deviation (SD) unless otherwise indicated. Statistical analyses were performed using GraphPad Prism software version 11.0.1 (GraphPad Software, San Diego, CA, USA). For comparisons among multiple experimental groups, one-way analysis of variance (ANOVA) followed by Tukey’s post hoc test was used. Because steatosis grade and inflammation grade represent ordinal histological scores, these variables were analyzed using non-parametric statistical tests. Differences among groups were evaluated using the Kruskal–Wallis test, followed by Dunn’s multiple comparison test when appropriate. A *p*-value < 0.05 was considered statistically significant.

## 3. Results

### 3.1. Effects of EtOH and 4HR on Body Weight and Glucose Homeostasis

**Figure 1:**
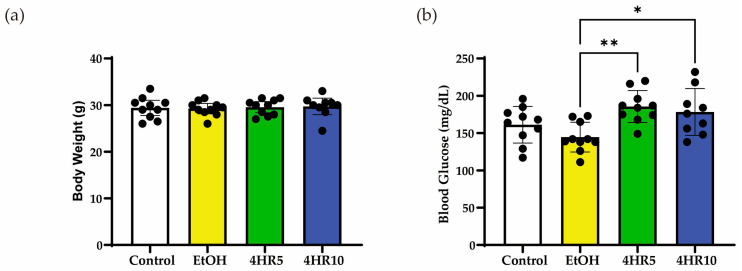
Effects of EtOH feeding and 4HR administration on body weight and glucose metabolism. (**a**) Final body weight of mice after the experimental period. (**b**) Fasting blood glucose (FBS) levels were measured after the EtOH feeding period. Data are presented as mean ± SD. Each dot represents an individual mouse (* *p* < 0.05, ** *p* < 0.01).

Body weight did not differ significantly among the experimental groups at the end of the study period (Figure 1a), indicating that neither chronic EtOH feeding nor 4HR administration markedly affected the overall growth or nutritional status of the animals. However, chronic EtOH consumption led to a significant reduction in fasting blood glucose levels compared to the control group (Figure 1b). Notably, 4HR administration partially normalized these levels, suggesting a restorative effect on basal glucose homeostasis.

### 3.2. Alterations in Glucose Tolerance and Insulin Secretory Capacity

**Figure 2:**
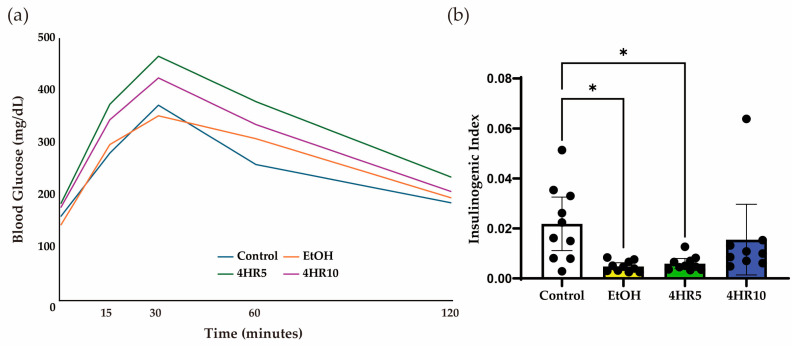
Effects of EtOH feeding and 4-hexylresorcinol administration on body weight and glucose metabolism. (**a**) Intraperitoneal glucose tolerance test (GTT). Blood glucose levels were measured at 0, 15, 30, 60, and 120 min after glucose administration. (**b**) Insulinogenic index calculated from glucose tolerance test data, reflecting early-phase insulin secretion. Data are presented as mean ± SD. Each dot represents an individual mouse (* *p* < 0.05).

The intraperitoneal glucose tolerance test (IPGTT) revealed distinct metabolic profiles across the experimental cohorts (Figure 2a). Following an exogenous glucose bolus, the EtOH-fed mice exhibited a blunted glucose excursion compared to controls, a trend that was partially reversed in the 4HR-treated group, particularly at 30 min after glucose loading. Compared with the EtOH group, the high-dose 4HR group showed a tendency toward improvement in the insulinogenic index, suggesting partial recovery of early-phase insulin secretory response; however, the values remained below those of the control group and the result should be interpreted cautiously (Figure 2b).

### 3.3. 4HR Attenuates EtOH-Induced Hepatic Inflammation

**Figure 3:**
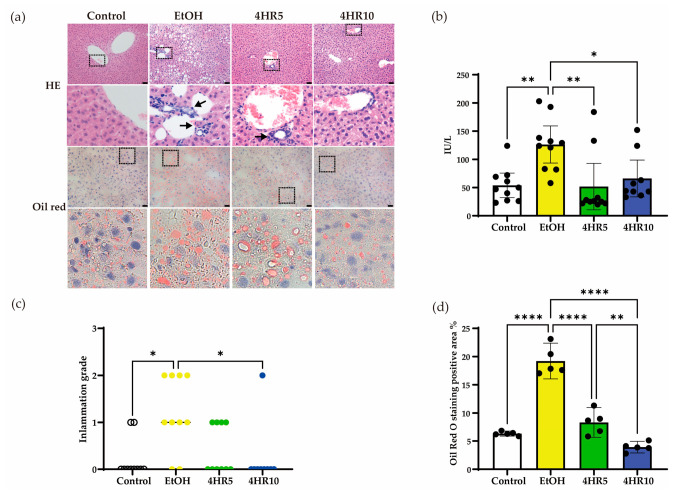
Effects of EtOH feeding and 4HR treatment on hepatic histological changes, lipid accumulation, and liver injury. (**a**) Representative histological images of liver sections stained with hematoxylin and eosin (H&E) and Oil Red O. Dashed boxes indicate regions shown at higher magnification in the lower panels. Arrows indicate representative inflammatory cell infiltration in the EtOH and 4HR5 groups. Scale bar = 50 μm. (**b**) Serum alanine aminotransferase (ALT) levels. (**c**) Inflammation grade based on histological evaluation of liver sections. (**d**) Quantification of Oil Red O–positive staining area in representative liver samples, demonstrating increased lipid accumulation in EtOH-fed mice. Data are presented as mean ± SD. Each dot represents an individual mouse (* *p* < 0.05, ** *p* < 0.01, **** *p* < 0.001).

Given the metabolic alterations observed in EtOH-fed mice (Figure 2), hepatic histology and biochemical markers of liver injury were further examined. Hematoxylin and eosin (H&E) staining of the control revealed preserved hepatic architecture with distinct cord-like arrangements of hepatocytes. In contrast, the EtOH-fed group showed marked hepatocellular vacuolation, a hallmark of hepatic steatosis (Figure 3a). Notably, administration of 4HR significantly mitigated these vacuolar changes, suggesting a protective effect against EtOH-induced structural damage.

To specifically evaluate the degree of lipid deposition, Oil Red O staining was employed. The EtOH-fed group showed prominent lipid droplet deposition within hepatocytes, which was markedly reduced in the 4HR-treated groups (Figure 3a). Quantitative morphometric analysis of the Oil Red O–positive areas confirmed a significant increase in lipid burden following EtOH consumption, an effect that was attenuated by 4HR treatment (Figure 3d).

Consistent with these histological findings, serum alanine aminotransferase (ALT) levels-a sensitive biochemical marker of hepatocellular necrosis-were significantly elevated in the EtOH-fed group compared to controls (*p* < 0.01; Figure 3b). 4HR administration significantly reduced ALT activities (*p* < 0.05), reinforcing the histopathological evidence of hepatoprotection. Furthermore, histological scoring of inflammatory activity revealed that EtOH-fed mice reached significantly higher inflammation grades, characterized by inflammatory cell infiltration. In contrast, 4HR treatment resulted in a substantial reduction in inflammation scores, indicating that 4HR suppresses the hepatic inflammatory response triggered by chronic ethanol exposure (Figure 3c).

### 3.4. EtOH-Induced Hepatic Glycogen Depletion Is Not Significantly Reversed by 4HR Treatment

**Figure 4:**
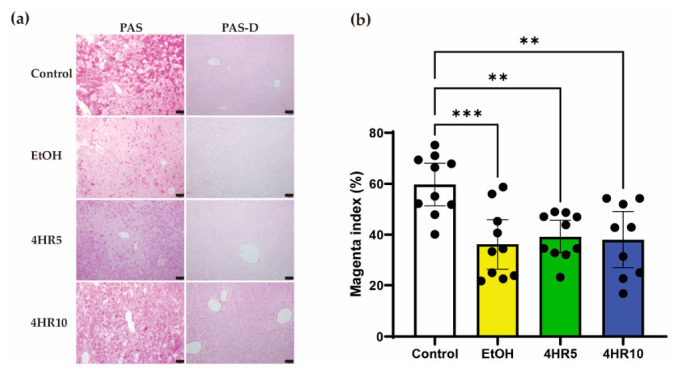
Effects of EtOH feeding and 4HR administration on hepatic glycogen content. (**a**) Representative liver sections stained with PAS and PAS after diastase digestion (PAS-D). PAS staining was markedly reduced in EtOH-fed mice compared with controls. Although PAS staining in the 4HR-treated groups appeared slightly increased by visual inspection, this qualitative tendency was not supported by quantitative analysis. Scale bar = 50 μm. (**b**) Quantification of PAS staining intensity expressed as the magenta index (%), representing the proportion of PAS-positive staining within the analyzed tissue area. Data are presented as mean ± SD, and each dot represents an individual animal. Statistical significance is indicated as shown (** *p* < 0.01, *** *p* < 0.005).

To further evaluate the impact of EtOH on hepatic energy substrates, hepatic glycogen content was assessed using PAS staining. In the control group, hepatocytes exhibited strong PAS-positive staining, indicating abundant glycogen storage (Figure 4a). In contrast, the EtOH-fed group showed markedly reduced PAS staining throughout the hepatic parenchyma, suggesting substantial depletion of hepatic glycogen stores. Although the 4HR-treated groups showed a slight qualitative tendency toward increased PAS staining compared with the EtOH group, this visual impression was not confirmed by quantitative analysis.

To confirm that the PAS signal reflected glycogen deposition, sections were subjected to diastase digestion (PAS-D) prior to staining. Diastase digestion effectively diminished PAS staining in all experimental groups, indicating that the observed PAS signal primarily represented glycogen deposition rather than other glycoproteins.

Quantitative morphometric analysis using the magenta index confirmed that EtOH feeding significantly reduced hepatic PAS-positive staining compared with the control group (Figure 4b). However, neither 4HR5 nor 4HR10 significantly increased the magenta index compared with the EtOH group. In addition, the 4HR10 group remained significantly lower than the control group. These findings indicate that, under the present experimental conditions, 4HR did not significantly restore EtOH-induced hepatic glycogen depletion.

### 3.5. 4HR Preserves Pancreatic Islet Cytoarchitecture and Attenuates Oxidative Stress

**Figure 5:**
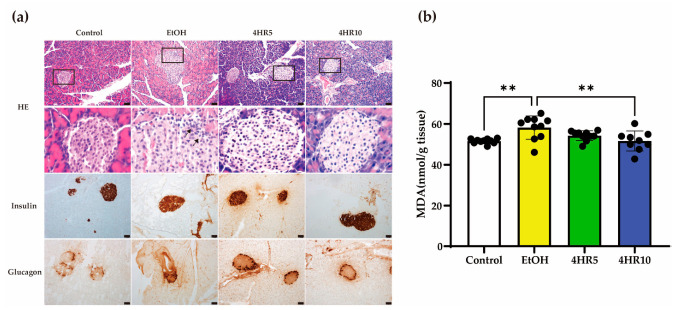
Effects of EtOH feeding and 4HR administration on pancreatic histology, endocrine hormone expression, and lipid peroxidation. (**a**) Representative pancreatic sections from the control, EtOH, 4HR5, and 4HR10 groups. H&E staining demonstrates pancreatic architecture and islet morphology. The boxed regions in the upper panels are presented at higher magnification in the corresponding middle panels. In the EtOH group, arrows indicate peri-islet inflammatory cell infiltration. Immunohistochemical staining for insulin identifies β-cells within pancreatic islets, whereas staining for glucagon identifies α-cells. Scale bars = 50 μm. (**b**) MDA levels in pancreatic tissue as an indicator of lipid peroxidation and oxidative stress. Data are expressed as nmol/g tissue. Values represent mean ± SD (** *p* < 0.01).

To determine whether chronic EtOH exposure affects pancreatic endocrine integrity, we performed histological and immunohistochemical (IHC) analyses. H&E staining of the control group revealed preserved pancreatic architecture, characterized by well-organized acinar cells and clearly defined islets of Langerhans (Figure 5a). In contrast, the EtOH-fed group showed marked disruption of the islet cytoarchitecture, featuring irregular borders and focal alterations in the surrounding exocrine parenchyma. Notably, 4HR administration partially but distinctly preserved the structural organization of the pancreatic islets.

IHC staining for insulin was performed to evaluate β-cell functional integrity. The control group displayed robust, uniform insulin immunoreactivity within the islet core. In contrast, EtOH exposure resulted in a substantial reduction in insulin-positive area and intensity, suggesting impaired β-cell secretory capacity. However, 4HR-treated mice exhibited significantly higher insulin immunoreactivity, suggesting partial preservation of β-cell functional integrity under EtOH exposure. Furthermore, the distribution of glucagon-positive α-cells, which was restricted to the islets’ periphery in controls, was deranged in the EtOH group. Treatment with 4HR effectively maintained a more typical α-cell topographic distribution, suggesting partial preservation of overall islet morphology.

To quantify the degree of oxidative damage, we measured malondialdehyde (MDA) levels in pancreatic tissue using the TBARS assay (Figure 5b). Chronic EtOH consumption induced a significant elevation in pancreatic MDA levels compared with the control group (*p* < 0.01), indicating exacerbated lipid peroxidation. Importantly, treatment with 10 mg/kg 4HR significantly reduced MDA levels (*p* < 0.01), whereas the 5 mg/kg dose failed to reach statistical significance (*p* > 0.05). These quantitative data corroborate the histological evidence, suggesting that attenuation of oxidative stress may contribute, at least in part, to the pancreatic effects observed with 4HR treatment.

### 3.6. 4HR Attenuates EtOH-Induced ER Stress in the Liver and Pancreas

**Figure 6:**
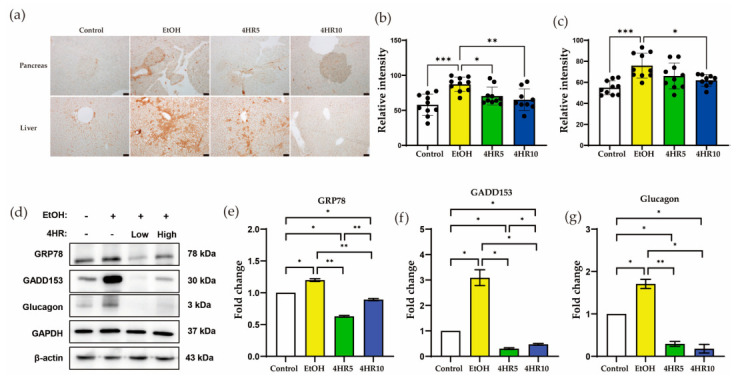
Effects of EtOH feeding and 4HR administration on endoplasmic reticulum (ER) stress–related protein expression in pancreatic and liver tissues. (**a**) Representative immunohistochemical staining of GADD153 in pancreatic and liver tissues from control, EtOH, 4HR5, and 4HR10 groups. Increased immunoreactivity was observed in EtOH-fed mice compared with the control group, while 4HR treatment reduced staining intensity. Scale bars = 50 μm. (**b**) Semiquantitative analysis of GADD153 immunostaining intensity in pancreatic tissue. (**c**) Semiquantitative analysis of GADD153 immunostaining intensity in liver tissue. (**d**) Western blot analysis of GRP78, GADD153 (CHOP), and glucagon expression in pancreatic tissue. EtOH exposure increased the expression of ER stress–related proteins compared with the control group, whereas 4HR treatment attenuated these changes. GAPDH was used as a loading control. (**e**–**g**) Quantification of Western blot results for GRP78 (**e**), GADD153 (**f**), and glucagon (**g**) normalized to GAPDH. Data are presented as mean ± SD. Statistical significance is indicated as shown (* *p* < 0.05, ** *p* < 0.01, *** *p* < 0.005).

To investigate whether the metabolic and histological alterations were associated with endoplasmic reticulum (ER) stress, we examined the expression of GADD153 (CHOP), a pro-apoptotic transcription factor and a marker of unresolved ER stress. Analysis was performed in both pancreatic and liver tissues by IHC and in pancreatic tissue by Western blotting. Immunohistochemical staining revealed minimal GADD153 immunoreactivity in the control group, whereas EtOH-fed mice showed markedly increased staining in both the pancreas and liver (Figure 6a). Quantitative analysis of GADD153 immunostaining confirmed a significant increase in the EtOH group compared with the control group in both pancreatic and liver tissues (Figure 6b,c). In pancreatic tissue, both 4HR5 and 4HR10 significantly reduced GADD153 immunoreactivity compared with the EtOH group, whereas in liver tissue, a significant reduction was observed in the 4HR10 group but not in the 4HR5 group (Figure 6b,c). Consistent with these findings, Western blot analysis in pancreatic tissue demonstrated increased expression of GRP78, GADD153 (CHOP), and glucagon in the EtOH group compared with the control group (Figure 6d). Quantitative densitometric analysis further supported these changes (Figure 6e–g). Quantitative analysis of pancreatic ER stress-related protein expression using β-actin as a complementary loading control showed results similar to those presented in Figure S2. Administration of 4HR reduced the expression of these ER stress-related markers in pancreatic tissue, although the degree of recovery varied among the individual markers.

## 4. Discussion

The present study showed that chronic EtOH exposure induced coordinated metabolic and tissue injury in both the liver and pancreas, including altered glucose homeostasis, hepatotoxic and inflammatory changes, depletion of hepatic glycogen, pancreatic islet disruption, and increased expression of the ER stress-related marker GADD153 (CHOP). These findings are broadly consistent with previous studies showing that chronic ethanol exposure causes metabolic and cellular injury in both the liver and pancreas [29]. Within this pathophysiologic context, the present results suggest that 4HR treatment may attenuate several EtOH-induced hepatic and pancreatic alterations in the same chronic ethanol-feeding model, supporting its potential as a multi-organ protective agent.

The hepatic alterations observed in the present study (Figure 3), including hepatocellular vacuolation, increased lipid deposition, and elevated serum ALT levels, are consistent with the characteristic features of alcohol-associated liver disease (ALD) [29]. Ethanol metabolism through alcohol dehydrogenase and cytochrome P450 2E1 generates acetaldehyde and ROS while shifting the intracellular redox state toward an elevated NADH/NAD^+^ ratio [30,31]. This metabolic imbalance suppresses fatty acid oxidation and promotes de novo lipogenesis, thereby contributing to the steatotic phenotype observed in the EtOH-fed group [32,33]. In the present study, 4HR treatment reduced hepatic vacuolar change, Oil Red O-positive lipid accumulation, and ALT elevation, suggesting that 4HR may attenuate hepatic injury associated with chronic ethanol exposure.

A striking finding of this study was the marked depletion of hepatic glycogen stores in EtOH-fed mice (Figure 4). While hepatic steatosis reflects the accumulation of energy substrates, the concurrent loss of glycogen indicates a disturbance in hepatic energy mobilization and glucose homeostasis. Ethanol metabolism increases the hepatic NADH/NAD^+^ ratio, which can suppress gluconeogenesis and alter hepatic glucose output, thereby contributing to fasting hypoglycemia and reduced glycogen storage [1,34,35]. In the present study, PAS staining in the 4HR-treated group appeared slightly greater than in the EtOH group by visual inspection (Figure 4); however, the quantitative analysis using the magenta index did not demonstrate a statistically significant recovery relative to the EtOH group. Therefore, the present data do not support the conclusion that 4HR restored hepatic glycogen reserves. This incomplete recovery may reflect the severity of chronic EtOH-induced disruption of hepatic energy metabolism, insufficient treatment duration or dose for glycogen restoration, or the possibility that 4HR primarily attenuates inflammatory, lipid-related, oxidative, and ER stress responses rather than directly normalizing hepatic glycogen storage. Nevertheless, our recent study demonstrated that 4HR enhanced hepatic GLUT4 expression, AMPK phosphorylation, and glucose uptake, and was associated with increased glycogen storage in hepatocytes in vivo [20]. Because AMPK is a key regulator of hepatic energy metabolism [36], these previous findings suggest that 4HR may influence hepatic glucose handling and metabolic adaptation under stress conditions, although the precise molecular mechanism was not directly established in the current chronic EtOH model.

Beyond the liver, our data underscore the high vulnerability of the pancreas to chronic EtOH exposure. The reduction in insulin immunoreactivity and the diminished insulinogenic index (Figure 2 and Figure 5) suggest impairment of pancreatic β-cell function. Because β-cells possess a limited endogenous antioxidant capacity, they are particularly susceptible to ROS and acetaldehyde generated during EtOH metabolism [37,38]. In the present study, chronic EtOH exposure was also associated with upregulation of GADD153 (CHOP) in both the liver and pancreas (Figure 6). CHOP is a well-established downstream mediator of unresolved ER stress and is induced when the unfolded protein response shifts from adaptive signaling toward pro-apoptotic pathways [39]. Therefore, the increased CHOP expression observed in the EtOH group is consistent with the possibility that chronic ethanol exposure triggered maladaptive ER stress in both organs. Notably, 4HR treatment reduced CHOP expression in the liver and pancreas (Figure 6). Although the present study did not directly assess upstream ER stress regulators, this reduction suggests that 4HR may attenuate EtOH-induced cellular stress and thereby limit progression to ER stress-associated apoptotic signaling.

The protective effects of 4HR observed in this study may be related to its previously reported antioxidant and cytoprotective properties. 4HR is a phenolic compound that has been widely used as an antimicrobial agent [40], but increasing evidence suggests that it can also modulate cellular stress responses and metabolic signaling pathways [20]. Phenolic compounds are known to act as free radical scavengers and can reduce oxidative damage by neutralizing ROS [41,42]. In experimental models, 4HR has been shown to attenuate oxidative stress–induced cellular injury and influence metabolic signaling pathways associated with energy homeostasis [43,44]. Because chronic EtOH exposure generates substantial oxidative stress and disrupts intracellular redox balance [45], the ability of 4HR to modulate these stress responses may contribute to its protective effects. In the present study, 4HR treatment was associated with improvement in several EtOH-induced alterations, including hepatic inflammation, pancreatic endocrine disruption, and GADD153 expression (Figure 2, Figure 3, Figure 4, Figure 5 and Figure 6). However, the glycogen-related findings should be interpreted more cautiously, because the quantitative analysis did not demonstrate statistically significant recovery relative to the EtOH group (Figure 4). Taken together, these findings support the possibility that 4HR may modulate metabolic and cellular stress responses under conditions of EtOH-induced injury, potentially through effects related to oxidative stress and ER stress-associated signaling. Although the precise molecular mechanisms remain to be clarified, the present results indicate that 4HR may have broader biological activities beyond its traditional antimicrobial role and may represent a candidate compound for mitigating EtOH-induced metabolic and tissue injury.

Despite the findings of this study, several limitations should be considered. First, although hepatic lipid accumulation was demonstrated by Oil Red O staining, this analysis was performed on selected frozen samples rather than the entire specimen set because frozen tissues were not available for all animals. Second, although the present study demonstrated increased expression of ER stress–related markers, including GADD153 (CHOP) and GRP78, key upstream signaling pathways such as PERK–eIF2α–ATF4 or IRE1α signaling were not evaluated, which would allow a more comprehensive characterization of ER stress activation. Third, although the results suggest that oxidative stress may contribute to EtOH-induced liver and pancreatic injury, direct measurements of ROS or antioxidant enzyme activity were not performed in this study. Fourth, this study did not include a 4HR-alone treatment group. Because 4HR itself may modulate metabolic signaling and cellular stress responses, inclusion of a 4HR-only group would have allowed a clearer distinction between its intrinsic biological effects and its protective effects against EtOH-induced injury. Fifth, although ER stress-related changes were assessed by immunohistochemistry in both organs, Western blot analysis was performed only in pancreatic tissue and not in liver tissue, which limited tissue-specific molecular comparison. Finally, the present study was conducted in a murine chronic EtOH-feeding model, and the findings should therefore be interpreted cautiously in relation to human disease. Although this model reproduces selected features of alcohol-induced metabolic and tissue injury under controlled conditions, it does not fully capture the complexity of human alcohol-related disorders, which are influenced by heterogeneous drinking patterns, nutritional status, sex differences, genetic background, comorbidities, and long-term clinical progression. In addition, 4HR administration was initiated concurrently with EtOH exposure in the present experimental design. This differs from the clinical situation of chronic alcohol use, in which hepatic and pancreatic damage may already be present before any potential intervention is started. Therefore, the present findings should be interpreted primarily as evidence of preventive or concurrent protective effects under controlled experimental conditions, rather than as therapeutic reversal of pre-existing alcohol-induced liver or pancreatic injury. Accordingly, the protective effects of 4HR observed in this study cannot be directly extrapolated to human therapeutic efficacy. Future studies should therefore include more comprehensive molecular analyses, additional oxidative stress-related markers, 4HR-only control groups, delayed-treatment models in which 4HR is administered after alcohol-induced tissue injury has been established, and complementary cellular and preclinical models to better define the mechanisms and translational relevance of 4HR in alcohol-associated metabolic and organ injury.

## 5. Conclusions

In conclusion, the present study demonstrates that chronic EtOH exposure induces coordinated metabolic and cellular alterations in both the liver and pancreas. EtOH feeding resulted in significant metabolic disturbances, including impaired glucose regulation, hepatic steatosis, and depletion of hepatic glycogen stores. In addition, EtOH exposure disrupted pancreatic endocrine integrity and increased the expression of the ER stress marker GADD153 in both liver and pancreatic tissues, indicating activation of stress-related cellular pathways. Importantly, administration of 4HR partially attenuated several EtOH-induced alterations, including hepatic lipid accumulation, pancreatic endocrine disruption, oxidative stress, and ER stress activation; however, 4HR did not significantly restore EtOH-induced hepatic glycogen depletion under the present experimental conditions. These findings suggest that EtOH-induced metabolic imbalance involves both hepatic and pancreatic dysfunction and that ER stress may play an important role in this process. The ability of 4HR to mitigate these changes indicates that it may exert protective effects by modulating metabolic and cellular stress responses. Collectively, this study highlights the potential role of 4HR as a protective agent against EtOH-induced metabolic and tissue injury, although further investigations are required to clarify its molecular mechanisms and therapeutic applicability.

## Figures and Tables

**Table 1:** Inflammation grade for liver tissue.

Grade	Histologic Criteria	Typical Appearance
0	No inflammatory cells	Normal liver parenchyma
1	Fewer than 2 inflammatory foci per 200× field	Occasional sinusoidal leukocytes
2	2–4 inflammatory foci per 200× field	Small clusters of inflammatory cells
3	More than 4 inflammatory foci per 200× field	Diffuse inflammatory infiltration

## Data Availability

The original contributions presented in this study are included in the article/Supplementary Material. Further inquiries can be directed to the corresponding authors.

## Supplementary Materials

The following supporting information can be downloaded at: https://www.mdpi.com/article/10.3390/biomedicines14051077/s1. Figure S1: Cumulative food intake; Figure S2: Quantitative analysis of pancreatic ER stress-related protein expression using β-actin as a complementary loading control.

## Abbreviations

The following abbreviations are used in this manuscript:

4HR	4-Hexylresorcinol
ALT	Alanine aminotransferase
ANOVA	Analysis of variance
AUC	Area under the curve
BCA	Bicinchoninic acid
CHOP	C/EBP homologous protein
CYP2E1	Cytochrome P450 2E1
DAB	3,3′-Diaminobenzidine
ECL	Enhanced chemiluminescence
ELISA	Enzyme-linked immunosorbent assay
ER	Endoplasmic reticulum
EtOH	Ethanol
GADD153	Growth arrest and DNA damage-inducible protein 153
GAPDH	Glyceraldehyde-3-phosphate dehydrogenase
GRP78	Glucose-regulated protein 78
GTT	Glucose tolerance test
H&E	Hematoxylin and eosin
MDA	Malondialdehyde
PAS	Periodic acid-Schiff
PBS	Phosphate-buffered saline
PVDF	Polyvinylidene difluoride
RIPA	Radioimmunoprecipitation assay
ROI	Region of interest
ROS	Reactive oxygen species
SD	Standard deviation
SDS-PAGE	Sodium dodecyl sulfate-polyacrylamide gel electrophoresis
STZ	Streptozotocin
TBA	Thiobarbituric acid
TBARS	Thiobarbituric acid reactive substances
