# Supplementary material for: 4-Hexylresorcinol Attenuates Ethanol-Induced Hepatic and Pancreatic Injury by Modulating Metabolic Dysfunction and Endoplasmic Reticulum Stress
Source: Biomedicines. 2026 May 9;14(5):1077. doi: 10.3390/biomedicines14051077 (PMC13204918; doi:10.3390/biomedicines14051077)

4-Hexylresorcinol Attenuates Ethanol-Induced Hepatic and Pancreatic Injury by Modulating Metabolic Dysfunction and Endoplasmic Reticulum Stress

Horațiu Rotar [1], Soon-Young Kang [2], Hyun-Seung Kim [2], Seung-Ki Hong [2], Yoon-Jo Lee [2], Ji-Hyeon Oh [2,*], Suyeon Park [3], Jongho Choi [3], Xiangguo Che [4], Seong-Gon Kim [2,*] and Je-Yong Choi [4]

1 Department of Oral and Maxillofacial Surgery and Implantology, Faculty of Dental Medicine, "Iuliu Hatieganu" University of Medicine and Pharmacy, 400012 Cluj-Napoca, Romania; dr.horatiu.rotar@gmail.com

2 Department of Oral and Maxillofacial Surgery, College of Dentistry, Kangwon National University, Gangneung 25457, Republic of Korea; syk980208@naver.com (S.-Y.K.); dannyk0314@naver.com (H.-S.K.); weuuhh@gwnu.ac.kr (S.-K.H.); _yoonjo@gwnu.ac.kr (Y.-J.L.)

3 Department of Oral Pathology, College of Dentistry, Kangwon National University, 7 Jukheon-gil, Gangneung 25457, Republic of Korea; sypark9101@gwnu.ac.kr (S.P.); jhchoi@gwnu.ac.kr (J.C.)

4 Department of Biochemistry and Cell Biology, Cell and Matrix Research Institute, School of Medicine, Kyungpook National University, Daegu 41944, Republic of Korea; xiangguo0622@naver.com (X.C.); jechoi@knu.ac.kr (J.-Y.C.)

* Correspondence: oms@gwnu.ac.kr (J.-H.O.); kimsg@gwnu.ac.kr (S.-G.K.); Tel.: +82-33-640-2468 (S.-G.K.)

Supplementary Figure S1. Cumulative food intake.

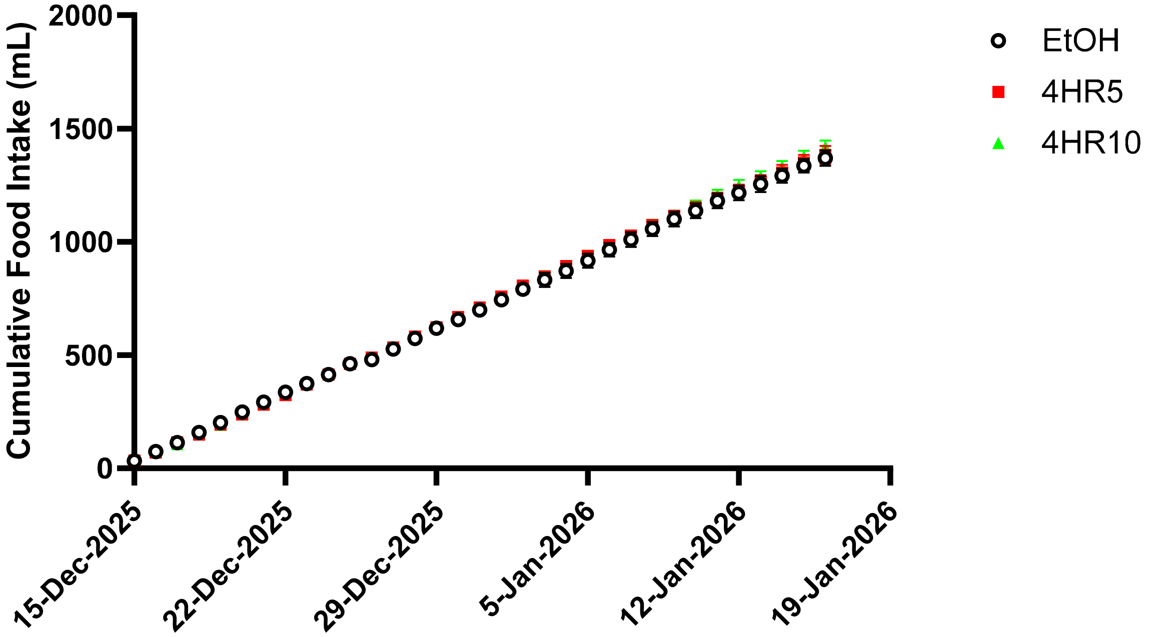

Repeated-measures two-way ANOVA demonstrated that cumulative food intake increased significantly over time in all groups. The effect of time was highly significant $F(32, 384) = 14131.02$, $p < 0.0001$, indicating a progressive increase in cumulative food consumption throughout the experimental period. In contrast, there was no significant overall effect of group $F(2, 12) = 0.65$, $p = 0.5402$, suggesting that total cumulative food intake did not differ significantly among the experimental groups. In addition, the interaction between time and group was not significant $F(64, 384) = 2.11$, $p = 0.1161$, indicating that the pattern of increase in cumulative food intake over time was comparable across groups. These findings suggest that, although cumulative food intake rose steadily during the study period, the treatments did not significantly alter either the overall amount of food consumed or the temporal pattern of food intake. The significant subject effect $F(12, 384) = 47.16$, $p < 0.0001$ further indicates that the repeated-measures matching was effective and that inter-subject variability was appropriately accounted for in the analysis.

Supplementary Figure S2. Quantitative analysis of pancreatic ER stress-related protein expression using β-actin as a complementary loading control. (a) Densitometric analysis of GRP78 expression normalized to β-actin. (b) Densitometric analysis of GADD153 (CHOP) expression normalized to β-actin. Relative expression levels are shown for the control, EtOH, 4HR5, and 4HR10 groups. Data are presented as mean ± SD. Statistical significance is indicated as shown (* $p < 0.05$).

(a)

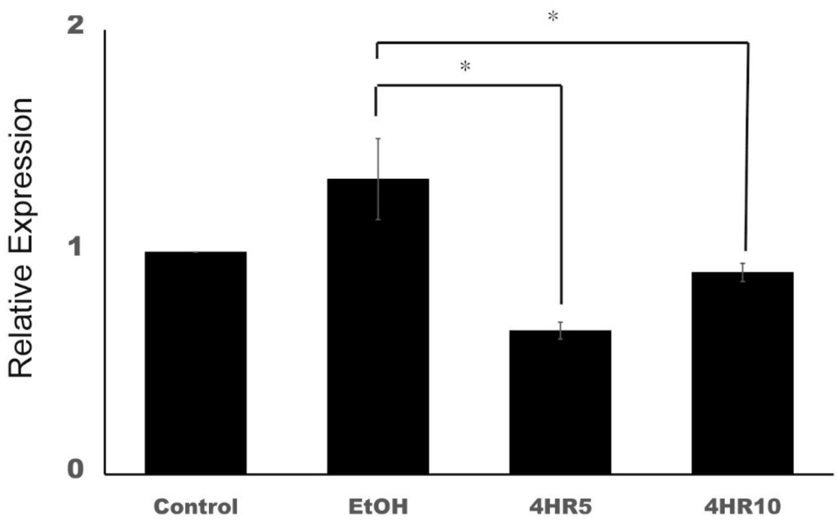

(b)

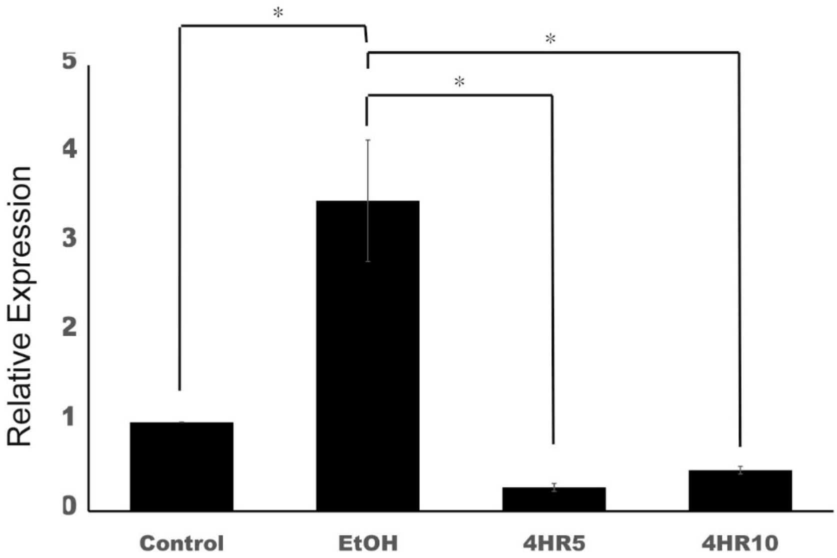

Supplement: Supplementary file 1 [file biomedicines-14-01077-s001.zip › biomedicines-4258549-supplementary.pdf]
